# Gross hematuria in nonagenarians admitted to a urological ward: prevalence, predictors, and outcomes

**DOI:** 10.1007/s00345-025-05975-w

**Published:** 2025-10-16

**Authors:** Andreas Banner, Magdalena Schneider, Stephan Madersbacher, Igor Grabovac

**Affiliations:** 1 Department of Urology, Klinik Favoriten, Kundratstrasse 3, Vienna, A-1100 Austria; 2 Department of Social and Preventive Medicine, Centre for Public Health, Medical University of Vienna, Vienna, Austria; 3 Sigmund-Freud Private University, Vienna, Austria

**Keywords:** Urology, Nonagenarians, Hematuria, Geriatrics, Frailty, Anticoagulation

## Abstract

**Purpose:**

To describe the trajectories of nonagenarians admitted to a urological ward and to identify risk factors for admission due to gross hematuria (GH) and its impact on discharge status.

**Methods:**

A retrospective cohort study of nonagenarians admitted to a tertiary care center between 2014 and 2022 was conducted. Data on demographics, comorbidities, medication, and outcomes were collected. Frailty was assessed via the Canadian Study of Health and Aging Clinical Frailty Scale (CSHA-CFS). Multivariable Poisson regression was used to assess risk factors for GH-related admissions. Secondary endpoints included changes in discharge status and survival, analyzed using Kaplan-Meier and restricted mean survival time (RMST).

**Results:**

Among 317 patients (median age 92 yrs [IQR 91–94], 63.7% male), 134 (42.3%) were admitted for hematuria. Antithrombotic therapy (aRR = 1.40, 95% CI 1.02–1.92, *p* = 0.013) and history of bladder cancer (aRR = 1.46, 95% CI 1.08–1.96, *p* = 0.013) were significantly associated with hematuria-related admissions. Increased social service utilization was more frequent among hematuria patients (13.4% vs. 8.7%), reaching significance in sensitivity analysis (aRR = 2.17 95%CI 1.16–4.06, *p* = 0.02). One-year mortality was higher among hematuria patients (34.3% vs. 24.5%), with lower RMST (12 vs. 15 months). In-hospital mortality during the index admission was low across both groups. The CSHA-CFS had limited discriminative power to predict 1-year mortality (AUC = 0.59).

**Conclusion:**

GH is a common reason for admission in nonagenarians and may indicate broader vulnerability. Geriatric assessment tools could aid in clinical decision-making and discharge planning. Future research should validate frailty-based risk stratification and address functional decline related to hospitalization in this population.

**Supplementary Information:**

The online version contains supplementary material available at 10.1007/s00345-025-05975-w.

## Introduction

In the European Union, the 85 + age group is the fastest-growing segment of the population. In Austria, with a population of 9.2 million, the number of nonagenarians is projected to increase by 175%, from 82,000 in 2010 to 227,000 by 2050 [1]. Many urological diseases, such as benign prostatic hyperplasia (BPH), bladder cancer (BC), and urinary incontinence, peak in incidence after age 80, posing a significant challenge for the future organisation of urological care [2–4].

Gross hematuria (GH) is a common symptom among elderly patients admitted to urological wards, often linked to underlying pathologies such as bladder and prostate cancer, BPH, or urinary tract infections, all of which increase with age [5]. The occurrence of GH may also be influenced by anticoagulant (OAC) or antiplatelet therapy, which are commonly prescribed for age-related conditions such as atrial fibrillation and cardiovascular disease (CVD) [6, 7]. A recent study from our group reported that almost half of all admissions of nonagenarians to a urological ward were due to GH [8]. Hospital admissions and subsequent invasive procedures can strain the cognitive function of elderly patients and may result in physical decline due to iatrogenic factors, such as immobilization associated with the use of drainage catheters [9]. This decline may further compromise autonomy and even affect discharge status (e.g., returning home or to a nursing home), as it increases the need for care resources [10]. Therefore, identifying risk factors for GH in nonagenarians offers a vital opportunity to improve care and support elderly patients’ autonomy.

Geriatric screening tools are easily available and have been shown to be associated with survival and clinical outcomes in elderly patients [11–13]. The Canadian Study of Health and Aging Clinical Frailty Scale (CSHA-CFS) is a validated 7-point global clinical judgement scale (1 = very fit to 7 = severely frail) that synthesizes baseline function, comorbidity, and cognition and can be completed in under two minutes. In prior work from our department, a CSHA-CFS >6 was associated with worse survival in nonagenarian patients [8].

To support clinical decision-making in this vulnerable population, identifying risk factors for GH-related hospitalizations and their downstream consequences may help improve patient-centered care. In this study, we provide an updated analysis of nonagenarians admitted to a urological ward, with a focus on predictors of GH-related admissions, changes in discharge status, and the prognostic value of frailty using the CSHA-CFS Given the growing relevance of geriatric assessments in urological practice, our findings aim to inform clinical decision-making and resource planning for an aging population.

## Patients and methods

All patients ≥90 years old who were admitted to our urological ward between 01.01.2014 and 31.12.2022 were identified. This timeframe was chosen based on data availability. After the removal of duplicate data, we retrospectively extracted the clinical history, demographics, comorbidities, medications at the time of admission. The CSHA-CFS was retrospectively defined on the basis of a chart review; the full instrument is described elsewhere [11]. The reason for and duration of hospital admission, as well as the urological procedures performed, were also recorded.

The primary endpoint was to identify risk factors (age, sex, antithrombotic therapy [anticoagulation and/or antiplatelet therapy], indwelling catheters, and a history of BC) for hospital admissions with GH in a cohort of nonagenarians. For sensitivity analyses, two definitions of GH were included: (i) GH documented as the reason for admission and (ii) GH requiring continuous bladder irrigation, clot evacuation or endoscopic treatment.

The secondary endpoints were to investigate changes in social service utilization and discharge outcomes – such as survival status, catheter dependence, and discharge destination (e.g., home vs. nursing home) – based on the reason for admission (hematuria vs. no hematuria). Social services were categorized as either home-based (e.g., scheduled community nursing or 24/7 community care) or facility based (nursing homes). Changes in social service utilization were defined as either a stepwise increase at discharge compared to baseline, based on a hierarchical scale (with 24/7 community nursing considered a higher level than scheduled care, and nursing home discharge representing a higher level of dependency than home-based services), or the new initiation of social services at discharge. The model was adjusted for CSHA-CFS, age, sex, medications, and a history of cardiovascular disease, chronic pulmonary obstruction, or stroke. To further investigate the impact of intensified treatment for GH, a sensitivity analysis was conducted, incorporating both definitions of the hematuria group as previously mentioned.

### Statistical analysis

We performed initial data analysis to identify missing or inconclusive values. Categorical variables are reported as counts and percentages, whereas continuous variables are reported as medians with interquartile ranges (IQRs). Group differences were assessed using the chi-square test for categorical variables and the Mann-Whitney U test or the t-test for non-parametric and parametric continuous variables, respectively. Survival curves were generated using the Kaplan‒Meier method, and follow-up distribution was summarized using the reverse Kaplan-Meier method. Group differences in Kaplan-Meier estimates were assessed using the log-rank test.

For both the primary and secondary endpoints, multivariable Poisson regression with robust standard errors was used to estimate adjusted risk ratios (aRRs) with corresponding 95% confidence intervals (95% CI). Model assumptions were assessed graphically, including the linearity of the continuous covariates.

To investigate survival, Cox proportional hazards models were used. Due to suspected violations of proportional hazards for CSHA-CFS, restricted mean survival time (RMST) was estimated. RMST was truncated at 20 months to align with the median follow-up and avoid overestimation. Both adjusted and unadjusted models were calculated, accounting for age, sex, CSHA-CFS and comorbidities (CVD, chronic pulmonary obstructive disease, hypertension, and stroke).

Receiver operating curves (ROC) analysis of the CSHA-CFS score was conducted to assess its discriminative ability and to identify an optimal dichotomization threshold. A CSHA-CFS score ≥ 5 was selected for stratified analyses.

All the statistical tests were conducted at a one-sided significance level of 0.05. Analyses were conducted using R version 4.4.1 (R Foundation for Statistical Computing, Vienna, Austria) with the packages ggsurvfit (v1.1.0), gtsummary (v2.0.2), survival (v3.8-3), survminer (v0.5.0) and survRM2 (v1.0-4).

Institutional review board approval was obtained (EK 24-152-VK), and the investigations were conducted in accordance with the Declaration of Helsinki.

## Results

### Patients

A total of 317 nonagenarians admitted between 2014 and 2022 were included. The median age was 92yrs (IQR 91–94) and 200 of 314 patients (63.7%) were male. Most admissions (84.5%) were emergency, while elective cases accounted for 15.5%. CSHA-CFS scores were similar between emergency (median 6, IQR 5–7) and elective (median 5, IQR 4–6) admissions. Median hospital stay was 5 days overall (IQR 3–8), with 5 days for emergency and 4 days (IQR 2–6) for elective admissions. Additional baseline characteristics and admission details are presented in Tables 1 and 2.

**Table 1:** Baseline characteristics of the study population, stratified by hematuria status

Characteristics	No Hematuria, *N* = 183 (57.7%)	Hematuria, *N* = 134 (42.3%)	*p*-value
Sex			0.351
m	111 (60.7%)	89 (66.4%)	
w	72 (39.3%)	45 (33.6%)	
Age at diagnosis (years)	92.0 (90.0–94.0)	92.0 (91.0–94.0)	0.238
CSHA-CFS	6.0 (5.0–6.0)	6.0 (5.0–7.0)	0.185
CSHA 0–4	41 (22.4%)	21 (15.7%)	0.177
CSHA ≥ 5	142 (77.6%)	113 (84.3%)	0.177
Therapies			
Number of medications	7.0 (5.0–9.0)	7.0 (5.0–9.0)	0.589
Antithrombotic therapy	112 (62.2%)	99 (74.4%)	**0.031**
Comorbidities			
Hypertension	149 (81.4%)	101 (75.4%)	0.245
Stroke	34 (18.6%)	27 (20.1%)	0.837
COPD	13 (7.1%)	14 (10.4%)	0.395
CVD	130 (71.0%)	111 (82.8%)	**0.022**
Past urological history			
Previous urological surgeries	54 (29.5%)	48 (35.8%)	0.286
Prostate cancer	18 (9.8%)	19 (14.2%)	0.311
Bladder cancer	21 (11.5%)	27 (20.1%)	**0.049**
Renal cell cancer	6 (3.3%)	6 (4.5%)	0.799
Voiding dysfunction	38 (20.8%)	35 (26.1%)	0.325
Reasons for emergency admission			
Urinary Tract Infection	91 (49.7%)	41 (30.6%)	**< 0.001**
Urinary Retention	30 (16.4%)	9 (6.7%)	**0.016**
Hydronephrosis	39 (21.3%)	18 (13.4%)	0.098
Urolithiasis	21 (11.5%)	7 (5.2%)	0.082

Data are presented as n (%) or median (IQR)

CSHA-CFS Canadian Study of Health and Aging Clinical Frailty Scale COPD Chronic Obstructive Pulmonary Disease CVD Cardiovascular Disease IQR Interquartile Range

**Table 2:** In-hospital procedures during the index admission and discharge outcomes, stratified by hematuria status

Characteristics	No Hematuria (*n* = 183)	Hematuria (*n* = 134)
Drainage catheter	19 (10.4%)	85 (63.4%)
Suprapubic catheter	5 (2.7%)	2 (1.5%)
Cystoscopy	11 (6.0%)	26 (19.4%)
Bladder evacuation	2 (1.1%)	13 (9.7%)
Bladder-TUR	21 (11.5%)	10 (7.5%)
Prostate-TUR	3 (1.6%)	4 (3.0%)
Insertion of ureteral stent	20 (10.9%)	4 (3.0%)
Percutaneous nephrostomy	4 (2.2%)	1 (0.7%)
Ureterorenoscopy	8 (4.4%)	2 (1.5%)
Circumcision	7 (3.8%)	6 (4.5%)
Discharge status		
Catheter in situ	66 (36.1%)	52 (38.8%)
Nursing home	55 (30.7%)	46 (35.7%)
Home	123 (67.2%)	83 (61.9%)
24/7 community nursing	16 (8.8%)	12 (9.0%)
Scheduled community nursing	29 (15.8%)	26 (19.4%)
Duration of admission (d)		
All admissions	5.0 (3.0–8.0)	5.0 (3.0–7.0)
Emergency	5.0 (3.0–8.0)	5.0 (3.0–7.0)
Planned	4.0 (3.0–6.0)	5.0 (1.0–5.0)

All data are presented as n (%) or median (IQR). Percentages may not sum to 100% due to rounding

TUR Transurethral Resection IQR Interquartile Range

## Gross hematuria

GH was the most common admission reason (*n* = 134, 42.3%). Compared to others, GH patients more often received antithrombotic therapy (74.4% vs. 62.2%) and had higher rates of CVD (82.8% vs. 71.0%) and BC (20.1% vs. 11.5%).

In multivariable Poisson regression, antithrombotic therapy (aRR = 1.40, 95% CI 1.02–1.92, *p* = 0.04) and a history of BC (aRR = 1.46, 95% CI 1.08–1.96, *p* = 0.01) were significantly associated with GH. Other variables, including age, sex and indwelling catheter status, were not significantly associated with GH (Table 3).

In a sensitivity analysis using an alternative GH definition, 85 patients were reclassified to the hematuria group and 232 to the no-hematuria group. No predictors reached statistical significance, although point estimates for antithrombotic therapy and bladder cancer aligned with the main analysis (Table 3).

**Table 3:** Adjusted risk ratios for hematuria-related admissions from Poisson regression: main and sensitivity analyses

	Main analysis			Sensitivity analysis		
**Characteristic**	**aRR** ^1^	**95% CI** ^1^	**p-value**	**aRR** ^1^	**95% CI** ^1^	**p-value**
Age	1.02	0.97–1.09	0.35	1.06	0.98–1.14	0.16
Sex (ref: Male)	0.92	0.69–1.21	0.53	0.75	0.50–1.14	0.18
Antithrombotic therapy	1.40	1.02–1.92	0.04*	1.36	0.88–2.08	0.16
Bladder cancer history	1.46	1.08–1.96	0.01*	1.12	0.67–1.84	0.67
Indwelling catheter use	1.09	0.82–1.46	0.55	1.08	0.72–1.62	0.70

^1^aRR = adjusted Risk Ratio, CI = Confidence Interval

## Interventions and discharge status

In total, 56 patients (17.7%) underwent at least one procedure under local anaesthesia, and 73 (23.0%) under general anaesthesia during the index admission. In the GH group, 63.4% received a drainage catheter, 19.4% underwent cystoscopy, and 9.7% had bladder evacuation under anaesthesia. Full details are in Table 2.

Indwelling catheter use increased from 27.1% at admission to 37.2% at discharge. A total of 34 patients (10.7%) experienced an increase in social service utilization at discharge. Among patients with GH, 13.4% required increased social support vs. 8.7% among other patients (aRR = 1.59, 95%CI 0.83–3.04; *p* = 0.16), indicating a non-significant association (Table 4).

**Table 4:** Adjusted risk ratios for increased social service utilization from Poisson regression: main and sensitivity analyses

	Main Analysis			Sensitivity Analysis		
**Characteristic**	**aRR** ^1^	**95% CI** ^1^	**p-value**	**aRR** ^1^	**95% CI** ^1^	**p-value**
Age	0.86	0.74–1.01	0.11	0.86	0.73-1.00	0.05*
Sex (ref: Male)	0.71	0.34–1.49	0.36	0.76	0.36–1.61	0.47
CSHA	1.00	0.81–1.25	0.98	0.99	0.81–1.22	0.94
Number of medications	0.96	0.85–1.08	0.47	0.95	0.84–1.07	0.41
Comorbidities						
CVD	0.86	0.38–1.96	0.72	0.87	0.39–1.94	0.74
COPD	1.71	0.66–4.39	0.27	1.97	0.76–5.12	0.16
Stroke	0.26	0.07–1.03	0.06	0.28	0.07–1.10	0.07
Hematuria	1.59	0.83–3.04	0.16	2.46	1.32–4.59	< 0.01*

^1^aRR = adjusted Risk Ratio, CI = Confidence Interval

COPD *Chronic Pulmonary Obstructive Disease* CSHA *Canadian Study of Health and Aging Frailty Scale* CVD *Cardiovascular Disease*

In a sensitivity analysis, which used an alternative definition of the hematuria group, increased social service utilization was observed in 16.4% of patients with GH vs. 8.0% of those without (aRR = 2.46, 95%CI 1.32–4.59, *p* < 0.01) (Table 4).

CSHA-CFS scores were included to adjust for baseline frailty. ROC analysis revealed poor discriminatory power (AUC = 0.46), so CSHA-CFS was included as a continuous variable in regression (Supplementary Fig. 2).

### Follow-up

The in-hospital mortality rate during the index admission was 2.2% (*n* = 3) in the GH group and 3.8% (*n* = 7) otherwise. Over a median follow-up of 20 months (95% CI: 16–27 months) (Supplementary Fig. 1), readmission occurred in 53.7% (*n* = 72) of GH patients vs. 53.6% (*n* = 98) of others. A single readmission occurred in 29.9% (*n* = 40) vs. 24.0% (*n* = 44); ≥2 readmissions in 23.9% (*n* = 32) vs. (*n* = 54).

ROC analysis of CSHA-CFS yielded an AUC of 0.59, indicating poor discrimination for one-year survival. Nonetheless, a cut-off of ≥ 5 was selected for stratification (Supplementary Table 3).

**Fig. 1:**
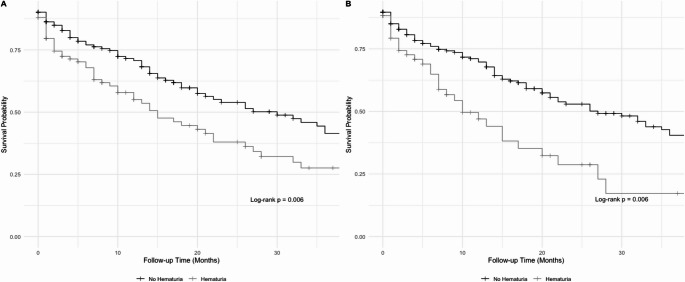
Kaplan–Meier survival curves stratified by hematuria-related admission status. (A) GH documented as the reason for admission. (B) GH requiring continuous bladder irrigation, clot evacuation or endoscopic treatment

One-year mortality was 34.3% in the hematuria group vs. 24.5% in others. Median survival was 15 months (95% CI: 10–26) for patients with hematuria and 30 months (95% CI: 20–38; *p* = 0.039) for others (Fig. 1). To avoid overestimation, RMST was estimated with truncation at 20 months.

Unadjusted RMST was 12 months (95% CI: 11–14) for hematuria and 15 months (95% CI: 13–16) otherwise. Differences in RMST and adjusted estimates could not be calculated due to data limitations, related to a substantial number of participants with no follow-up time. As exclusion would introduce bias, only unadjusted estimates are reported.

Sensitivity analyses using the alternative GH definition yielded essentially unchanged results for in-hospital mortality rates (2.2% vs. 3.8%) and readmission rates (57.7% vs. 52.2%). One-year mortality was 37.7% in GH vs. 25.4% in others. Median survival was 10 months (95% CI: 7–22) for patients with GH and 27 months (95% CI: 21–38; *p* = 0.006) for others (Fig. 1). Unadjusted RMST was 11 months (95% CI: 9–13) in GH patients compared to 14 months (95% CI: 13–16) in others.

## Discussion

The present study highlights the characteristics and outcomes of nonagenarians admitted to a urological ward over an eight-year period, with a particular emphasis on GH as the leading cause of hospitalization among geriatric urological patients. In line with previous research from the UK and our own earlier findings, GH accounted for over 40% of all admissions in this age group, reinforcing its relevance in geriatric urological care [8, 14]. Our study builds on previous work from our group by including a larger cohort and longer follow-up. It also adds adjusted analyses for frailty, social support, and survival.

GH-related admissions were significantly associated with antithrombotic therapy (aRR = 1.40, 95% CI 1.02–1.92) and a history of bladder cancer (aRR = 1.46, 95% CI 1.08–1.96). The elevated risk may reflect BC recurrence and previous treatment effects (e.g., intravesical instillations, irradiation), while antithrombotic use underscores the need to balance bleeding risk and cardiovascular protection, especially in frail patients [15–18]. Though causality cannot be inferred, both factors may help guide risk stratification and early intervention.

GH was not consistently associated with increased care dependency at discharge. In the primary model, increased social service utilization was more frequent among GH patients but did not reach statistical significance. However, sensitivity analysis using a more interventional GH definition (e.g., requiring continuous irrigation or clot evacuation) showed a significant association (aRR = 2.46, 95%CI 1.32–4.59). This likely reflects the broader clinical burden of GH management - such as prolonged catheterization, procedural interventions, and the associated risk of immobilization. Immobilization during hospitalization is a known driver of functional decline in older adults. A meta-analysis reported hospital-associated disability in approximately 30% of older patients, with immobility being one of the strongest contributors to new or worsening dependence at discharge [19]. Even short bed rest has been linked to sarcopenia and declines in strength and function in elderly patients [20, 21]. These effects may be especially relevant in frail individuals. GH patients in our cohort had slightly higher CSHA-CFS scores, suggesting greater baseline vulnerability. Although two-thirds were already receiving care services at admission - limiting the detectability of changes - our findings support frailty and in-hospital immobility as key contributors to post-discharge care needs.

Although in-hospital mortality was low in both groups, one-year mortality was higher in patients admitted with GH (34.3% vs. 24.5%), and RMST was shorter (12 vs. 15 months). These descriptive differences may reflect greater overall vulnerability in this subgroup. While the CSHA-CFS showed limited discriminatory accuracy in our cohort (AUC = 0.59), similar tools have been linked to mortality risk in older surgical and hospitalized populations, including those undergoing urological care [11–13]. Although our analysis was limited to unadjusted comparisons, these findings support further exploration of frailty-based screening to guide prognosis and shared decision-making in very old patients.

Polypharmacy was prevalent across both groups, with a median of seven medications (Table 1). While no direct associations were observed with clinical outcomes, prior studies have linked antithrombotic drug combinations and drug-drug interactions to prolonged bladder irrigation and increased bleeding events [22–24]. Our findings highlight the importance of comprehensive medication reviews - especially in frail, older patients presenting with GH - to minimize avoidable adverse events.

Approximately one-quarter of patients had an indwelling catheter at admission, increasing to over one-third at discharge. While long-term catheterization is a known risk factor for bladder cancer, in this context, it may also reflect advanced frailty or functional decline [25]. Invasive workup should be individualized, considering comorbidities, cognitive status, and patient preferences.

To efficiently identify patients at risk for geriatric impairments, rapid screening tools such as the G8 Geriatric Screening Tool and CSHA-CFS have been developed [11, 26]. Although direct comparisons across screening tools remain limited, the CSHA-CFS offers a pragmatic approach based on clinical judgment and has shown predictive value for mortality and functional decline in various settings [11, 12]. While comprehensive evidence on frailty screening in urological populations is still emerging, international urological guidelines recommend incorporating geriatric assessment tools to support individualized treatment decisions, particularly in older adults with prostate or bladder cancer [27–29]. Further standardization and validation of these tools in urology-specific contexts remain important priorities.

This retrospective, single-centre study has several limitations. Small subgroup sizes may have limited power, and findings may not generalize beyond our highly developed, free-access health-care setting. Survival analyses could not be adjusted for potential confounders due to data limitations and should be interpreted cautiously. We did not adjust for ethnicity or sex, which may influence GH presentation and outcomes. Antiplatelet and anticoagulant therapies could not be reliably distinguished in many cases and were combined; this may reduce generalizability, though prior work suggests only marginal additional risk with combined therapy. Only baseline laboratory results were available; serial hemoglobin values and transfusion data were not systematically recorded, precluding reporting of hemoglobin decline or transfusion rates. Finally, CSHA-CFS was assessed retrospectively for the index admission only, precluding time-dependent analyses of in-hospital change.

## Conclusions

Our findings emphasize the importance of recognizing gross hematuria as a prevalent and complex presentation among nonagenarians in urological care. Risk stratification should account for factors such as antithrombotic therapy and prior bladder cancer, which may help anticipate admissions and tailor early intervention strategies. While frailty screening using tools like the CSHA-CFS may support individualized decision-making, further work is needed to determine their optimal role and timing in urological settings. Clinical pathways should also consider the potential consequences of hospitalization - including immobilization and post-discharge care dependency - particularly in frail or socially vulnerable patients. Future research should prioritize prospective, multicenter designs with standardized frailty assessments and functional outcomes to improve prognostic accuracy and inform resource allocation. The integration of geriatric principles into routine urological practice will be essential to meeting the needs of an increasingly aged patient population.

## Supplementary Information

Below is the link to the electronic supplementary material.

Supplementary Material 1

## Data Availability

The datasets generated and/or analysed during the current study are not publicly available but are available from the corresponding author upon reasonable request.

## Abbreviations

95% CI: 95% confidence intervals
AF: Atrial fibrillation
DDI: Drug‒drug interaction
ECOG: Eastern cooperative oncology group
CSHA-CFS: Canadian study of health and ageing clinical frailty scale
CVD: Cardiovascular disease
GH: Gross hematuria
IQR: Interquartile range
OAC: Oral anticoagulation
OR: Odds ratio
SD: Standard deviation
TUR: Transurethral resection
UTI: Urinary tract infection
