# Supplementary material for: Gross hematuria in nonagenarians admitted to a urological ward: prevalence, predictors, and outcomes
Source: World J Urol. 2025 Oct 16;43(1):615. doi: 10.1007/s00345-025-05975-w (PMC12532713; doi:10.1007/s00345-025-05975-w)

**Gross hematuria in nonagenarians admitted to a urological ward: Prevalence, predictors, and outcomes**

**Running title: MHU 90+**

Andreas Banner^1,2^ and Magdalena Schneider^1^, Stephan Madersbacher^1,3^, Igor Grabovac^2^

^1^ Department of Urology, Klinik Favoriten, Vienna, Austria

^2^ Department of Social and Preventive Medicine, Centre for Public Health, Medical University of Vienna, Austria

^3^ Sigmund-Freud Private University, Vienna, Austria

Correspondence:

Andreas Banner, MD ([andreas.banner@gesundheitsverbund.at](mailto:Stephan.madersbacher@gesundheitsverbund.at))

Table of contents

Page 2 …………………………………………………….. Supplementary Fig. 1

Page 2 …………………………………………………….. Supplementary Fig. 2

Page 2 …………………………………………………….. Supplementary Fig. 3

Supplementary Figure 1. Follow-Up distribution estimated using the reverse Kaplan-Meier Method by the presence of hematuria in the index admission.


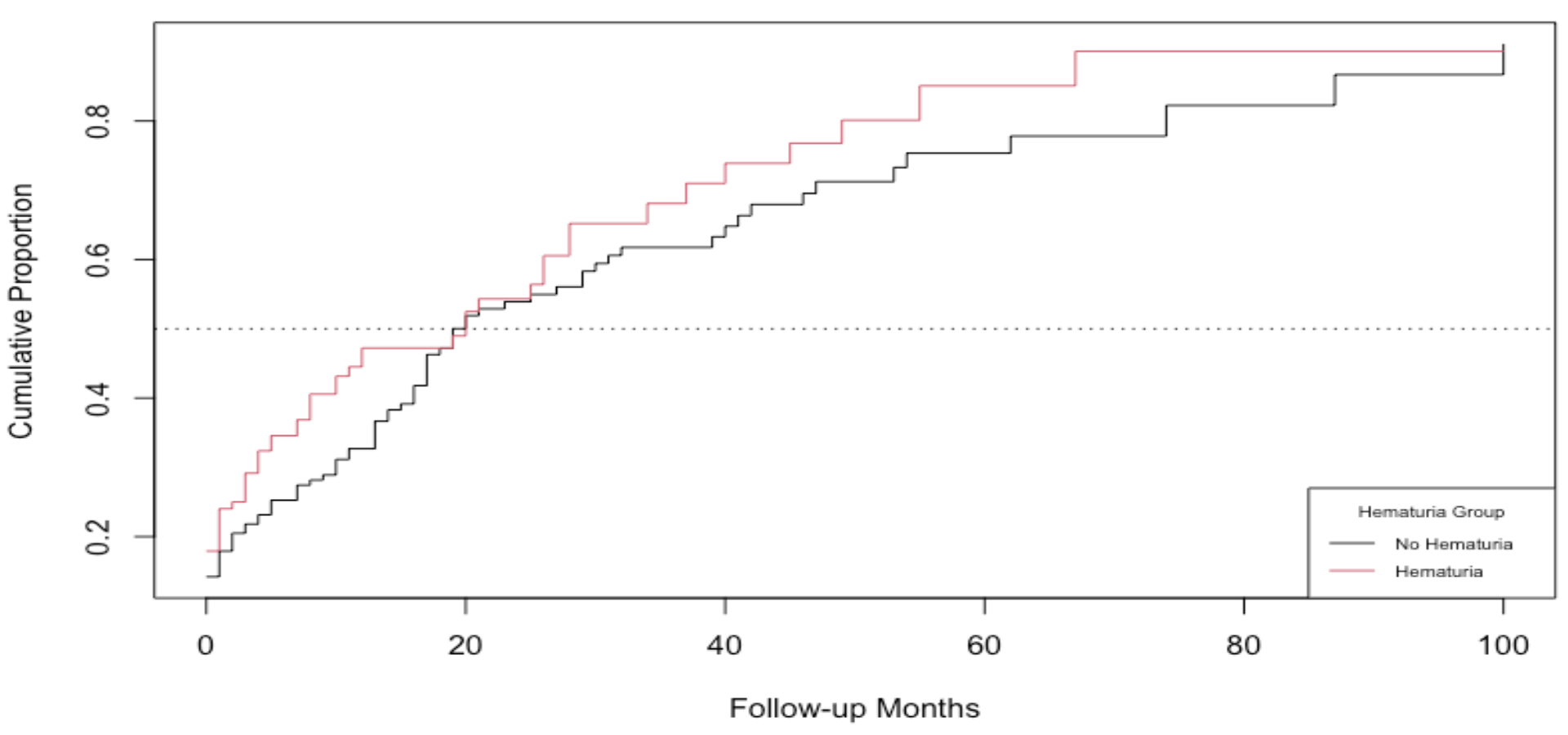


Supplementary Figure 2. Standard ROC Curve for CSHA as a Discriminator of Changes in Social Service Utilization (AUC=0.460)


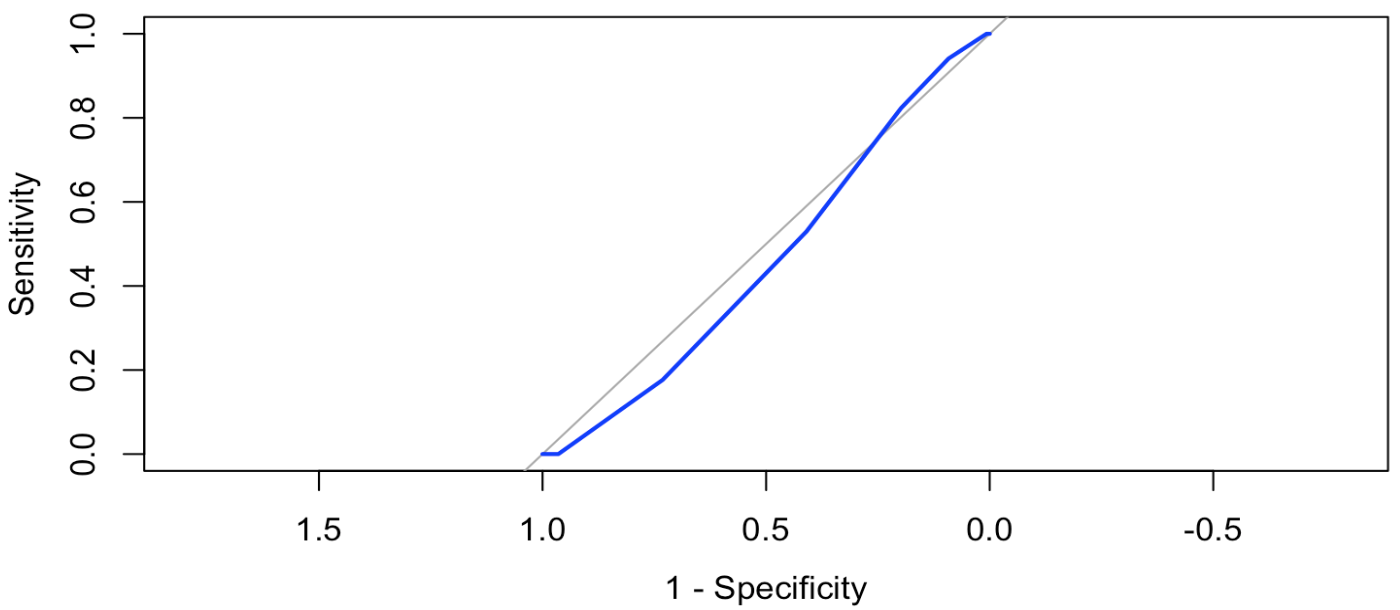


Supplementary Figure 3. Time-Dependent ROC Curve at 12 Months for CSHA and Social Service Utilization (AUC=0.592)


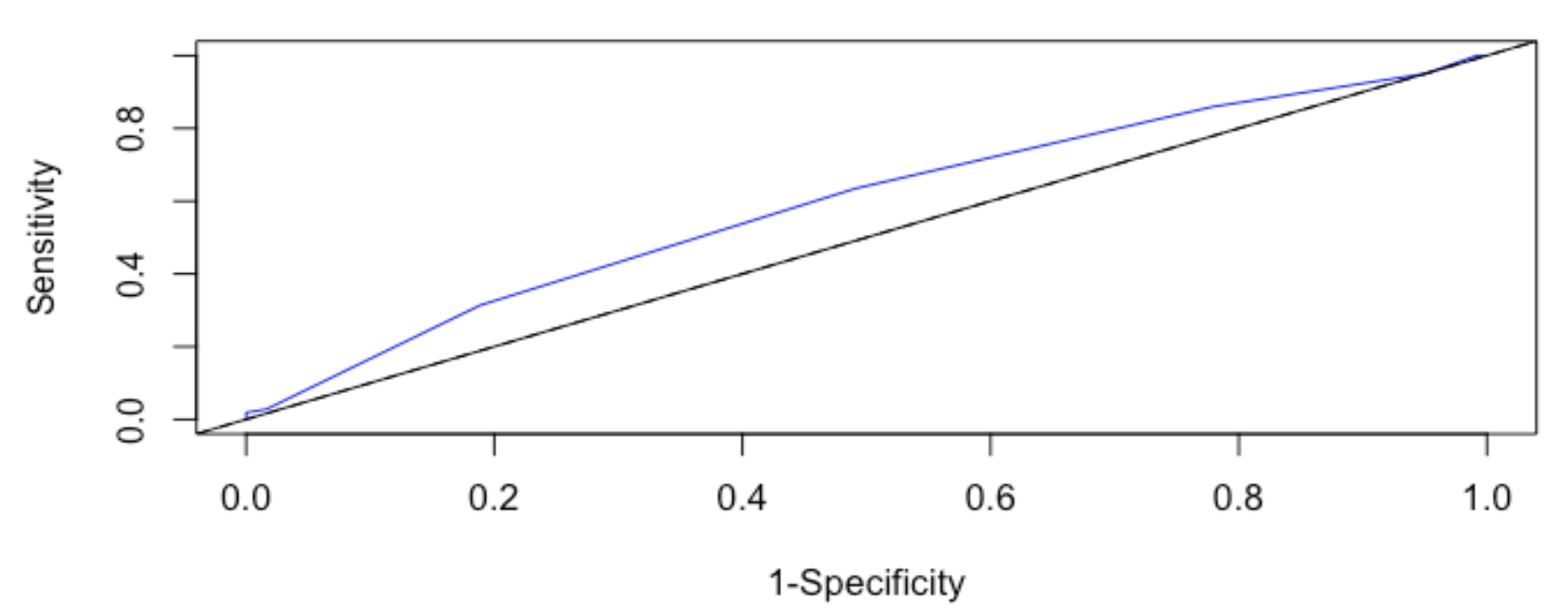

Supplement: Supplementary file 1 — Supplementary Material 1 [file 345_2025_5975_MOESM1_ESM.doc]
